# Gonorrhœa and Homœopathy

**Published:** 1891-01

**Authors:** S. A. Kimball

**Affiliations:** 124 Commonwealth Avenue; Boston, Mass.


### GONORRHŒA AND HOMŒOPATHY.

EDITORS OF THE HOMŒOPATHIC PHYSICIAN:

It will be a great disappointment if Dr. T. F. Allen fulfills his intention to refrain from expressing his views upon what a "consistent homœopathist" may or may not do. The pleasure ot reading and refuting his very peculiar ideas on the subject will then be lost, and it is hoped he will reconsider his decision.

In the December number of THE HOMŒOPATHIC PHYSICIAN, at page 548, in an article defending the suppression of gonorrhœa by injections, he says he has known of "one man suffering eighteen months continuously, though treated by a careful prescriber with the two-hundredth dilution." What of it? The man was undoubtedly in much better general health for having had his discharge so long a time under careful prescribing than if it had been suppressed by local measures within the first few weeks.

It would not have continued so long unless there was a psoric or sycotic condition in the patient's system, which, under the careful prescribing, was ridding itself of the psora or sycosis by means of the discharge.

Many patients are much more healthy after the strict homœopathic treatment of a gonorrhœal discharge that continues for a long time than ever before. The fact of having the discharge is not nearly so unfortunate as having it suppressed.

Young physicians, or older ones for that matter, should not be deterred from treating their gonorrhœal patients strictly homœopathically by this bugaboo of a discharge! Dr. Allen observes that physicians practicing pure Homœopathy treat fewer cases of this kind as the years pass on. How does he know this? It does not seem to obtain in this vicinity. Many cases still continue to come from allopathic hands after treatment by injections *secundem artem*, and the "damned spot" will not "out" except by careful homœopathic prescribing. Usually in such cases an increased discharge is noticed after the first appropriate remedy. How is that accounted for unless by the previous suppression? "Gonorrhœa may be considered a poison," says Dr. Allen. No doubt of it. What is that "poison" doing between the time of its infection and its local manifestation by a discharge? What is the poison of syphilis, scarlet fever, small-pox, diphtheria, or any contagious disease doing in the period of incubation but infecting the whole system, and at last appearing on the surface in its local manifestations?

And when the local antidotes (?) of the gonorrhœal poison, the syphilitic poison, the small-pox poison, etc., etc., are found, and the poisons are carefully washed away, what will there be left for the "consistent homœopathist"? Surely a sorry state of affairs!

Dr. Allen admits that iritis, suppurative nephritis, and "a host of bad things follow the injudicious treatment of the acute stage."

This hardly justifies him in following the same general method of suppression; and how does he know but that the same apparently "most satisfactory results" followed the "injudicious treatment," as he observes by persistent washing with his "little salt of soda or zinc or mercury."

The effects of suppressing a gonorrhœa do not appear the next day, month, or year necessarily, but they come at some future time, and if Dr. Allen does not see them, it does not invalidate in the slightest degree the testimony of those who do see them.

He says, " A homœopathic physician who determines to practice only Homœopathy must send away a great many patients to other doctors if he would do the best for them."

Shades of Hahnemann! A homœopathic physician must not practice Homœopathy if he would do the best for his patients! What shall he do, practice allopathy or eclecticism? It would then be much more consistent for the "consistent (?) homœopathist" to give up the name of homœopathist entirely, and sail under his true colors of eclecticism or allopathy. By their deeds ye shall know them.

Dr. Allen says, " Men *will not* tolerate a discharge for months when a safe washing will help them get well speedily." That may be his experience, but it is certainly not the experience of others.

When the dangers of suppressing the discharge are properly explained to the patient, such as the iritis and the suppurative nephritis that *Dr. Allen has seen,* and when the patient is told, as he should be, that the discharge may last four, six, or twelve months, for that matter, but that when he is cured that will be the end of it, most sensible men will prefer the cure to the suppression, especially if they have friends who have subjected themselves to the washing regime, and who are constantly breaking out with the same old case; those who have been through such an experience themselves are usually very easily convinced by the plain, unanswerable logic of the homœopathist.

The majority of people are not fools, they have much more respect for a physician who is conscientiously endeavoring to cure them, than for one who yields to their demand that the discharge must be stopped, and endeavors, often ineffectually, to stop it by local measures. A homœopathic physician never raises himself in his own esteem or in that of his patients by such proceedings. This is plainly seen by the constant attempts of those who do such things to explain or apologize for their departures from Homœopathy, and if their object is simply to hold the patient, logically they will not stop at washing out urethras.

"It is unnecessary to risk stricture, cystitis, nephritis, orchitis, rheumatism, etc., by properly washing out the discharge. What is the harm !!" says Dr. Allen. Well, in the first place, such things do follow the suppression of the discharge by washing out the urethra. Dr. Allen admits that he has seen serious effects, but claims that by *properly* washing out the urethra there is no harm. Does he know more about urethra washing than men in the allopathic school who have studied and followed that method of treatment for years, and who use the same salts of soda, zinc, and mercury? Undoubtedly they would tell you that they never see any bad effects, but Dr. Allen knows better. He uses the same arguments, the same salts, and claims the same result that there is no harm done, but homœopathists know better.

Worse results, of course, follow the suppression of a sycotic gonorrhœa than a simple urethritis, but no one can tell the difference in the first stages, and a physician who professes to be a homœopathist is no more justified in suppressing one than the other.

"Sores on the surface" are not cleansed by consistent homœopathists with salts of soda, zinc, or mercury. Probably Dr. Allen would claim that a "consistent homœopathist" could use carbolic acid or iodoform for their supposed antiseptic properties with perfect propriety. It is difficult to take his next statement seriously, "One may still be a consistent homœopathist and wash out or antidote by other means a gonorrhœal virus." Probably it was intended to be taken seriously, though it is difficult to understand how one in the full possession of his senses could imagine that others would look at it in that manner.

That one may be a consistent homœopathist and yet do things entirely inconsistent with the teachings of Hahnemann, the law of Homœopathy, or the dictates of common sense, seems very strange, but that any one would defend this inconsistency is stranger still. Where will it end? The same arguments that he uses for injections in gonorrhœa will apply to Quinine in malaria, Morphine in pain, or anything else that the prescriber sees fit to use under the guise of Homœopathy. No wonder allopathic physicians are disgusted at such subterfuges. Strict homœopathists laugh at these excuses of poisons, etc., as absurd. The so-called eclectic homœopathists hail with joy any additional excuse for eclecticism, but the pity of it is that the young beginner may be led by just such specious reasoning to try these same expedients, and they are often the means of his departing hopelessly from homœopathic truths.

In the November number of THE HOMŒOPATHIC PHYSICIAN for 1887, Dr. Allen gave us, in a very plausible manner, his views upon the use of Morphine, Quinine, washing out the urethra, local applications, etc., and he said that while he did not use such measures very often he used them whenever he thought them necessary. His views do not appear to have changed much since then, and it would be interesting to learn whether he uses these measures more frequently now than formerly. Such things were to be used whenever and as often as the necessity arose, and their use was to be governed simply by circumstances calling for them.

Now, if allopathic treatment is to be used whenever it seems necessary, and homœopathic measures are employed when *they* seem sufficient, it is perfectly proper to ask Dr. Allen, in the friendly manner that he wishes the discussion carried on, why he does not give up the title of homœopathist, which implies a certain strict method of practice that people expect who employ a homœopathic physician, and adopt that of eclectic, which covers all sorts of practices, and seems to exactly fit the case.

It would do away with the necessity of his constantly rising to explain procedures and endeavoring to reconcile the consistent with the inconsistent, and would be an inestimable boon to the progress of true Homœopathy.

S. A. KIMBALL,
124 Commonwealth Avenue.

BOSTON, MASS., December, 1890.

